# L-Asparaginase Exerts Neuroprotective Effects in an SH-SY5Y-A53T Model of Parkinson’s Disease by Regulating Glutamine Metabolism

**DOI:** 10.3389/fnmol.2020.563054

**Published:** 2020-09-30

**Authors:** Qingxi Zhang, Yuyuan Gao, Jiahui Zhang, You Li, Jianing Chen, Rui Huang, Guixian Ma, Limin Wang, Yuhu Zhang, Kun Nie, Lijuan Wang

**Affiliations:** ^1^The Second School of Clinical Medicine, Southern Medical University, Guangzhou, China; ^2^Department of Neurology, Guangdong Provincial People’s Hospital, Guangdong Academy of Medical Sciences, Guangdong Neuroscience Institute, Guangzhou, China

**Keywords:** L-asparaginase, Parkinson’s disease, α-synuclein, glutamine, metabolism therapy

## Abstract

**Background**: Parkinson’s disease (PD) is the second most common neurodegenerative disease worldwide and involves deficiencies in alpha-synuclein (α-Syn) degradation. Effective therapeutic strategies for PD are urgently needed. L-asparaginase (L-ASNase) has been developed for therapeutic applications in many fields because it catalyzes the hydrolysis of asparagine and glutamine in cancer cells, which may also activate autophagy and induce the degradation of accumulated α-Syn. However, the efficacy and related mechanism of L-ASNase in PD remain poorly understood.

**Methods**: We determined the correlation between L-ASNase and autophagic degradation of α-Syn in a cell model of PD. Mitochondrial function and apoptosis were examined in the presence or absence of L-ASNase. Then, we applied GC-MS/MS targeted amino acid metabolomics analysis to validate the amino acid regulation induced by L-ASNase treatment. Glutamine was added to verify whether the neuroprotective effect was induced by deprivation of glutamine. α-Syn-related autophagy and mitochondrial fusion/fission dynamics were detected to explore the mechanism of L-ASNase-based therapy in PD.

**Results**: L-ASNase activated the autophagic degradation of α-Syn in a cell model of PD without cytotoxicity at specific concentrations/times. Under these conditions, L-ASNase showed substantial neuroprotective effects, including improvements in mitochondrial function and decreased apoptosis. Through GC-MS/MS targeted analysis, glutamine metabolism was identified as the target of L-ASNase in PD treatment, and the neuroprotective effect of L-ASNase was reduced after glutamine supplementation.

**Conclusions**: Our study demonstrated for the first time that L-ASNase had a neuroprotective effect on a cell model of PD through a moderate deprivation of glutamine, which induced autophagic activation and mitochondrial fusion. Therefore, we demonstrated that L-ASNase could be a promising and effective drug for PD treatment.

## Introduction

Parkinson’s disease (PD), a progressive neurodegenerative disorder, is the second most common neurodegenerative disease worldwide (Tysnes and Storstein, 2017). To date, the levodopa (a dopamine precursor) regimen remains the primary treatment for PD, and the side effects include decreased efficacy, dyskinesia, and motor fluctuations after long-term use. Moreover, the current therapeutic regimen provides a limited improvement in overall survival, and drug sensitivity is decreased after a long period of use (Fahn, 2008). Therefore, the identification of other effective therapeutic targets and the development of new therapeutic strategies for PD are urgently needed.

Cell metabolism is an important field that can be linked to disease pathogenesis to elucidate the underlying mechanisms and identify potential therapeutic targets. Increasing evidence suggests that PD displays metabolic abnormalities, especially in nutritional metabolism (Yao et al., 2011; Anandhan et al., 2017). To date, mechanistic research on PD has mainly focused on lipid metabolism (Alecu and Bennett, 2019; Hallett et al., 2019; Xicoy et al., 2020). Another crucial nutrient, amino acids, has received little attention in PD research. Amino acids have numerous effects in the central nervous system, such as changes in neurotransmitters, neuromodulation, and regulation of energy metabolism (Fagg and Foster, 1983; Malik and Willnow, 2019). Amino acids have been increasingly shown to regulate key metabolic pathways that are necessary for cell maintenance and growth (Anthony and Gietzen, 2013). However, the relationship of abnormal amino acid metabolism and PD with neurodegeneration has not been fully elucidated.

The metabolic balance of amino acids has been widely shown to regulate autophagy in response to nutrition stress (Hietakangas and Cohen, 2009; Wu et al., 2015; van Niekerk et al., 2018). For example, autophagy could be induced by nutrient deprivation and inactivated by amino acids (van Niekerk et al., 2018). Autophagy is an essential catabolic mechanism that delivers misfolded or pathogenesis-related proteins to the lysosome for degradation. Defects in this degradation pathway can result in the accumulation of protein aggregates. Aggregated alpha-synuclein (α-Syn), a key pathogenic element of PD, was confirmed to be cleared by autophagy. When the autophagic mechanism is damaged or insufficient for the aggregated α-Syn, α-Syn aggregates will accumulate and contribute to neural degeneration (Xilouri et al., 2013, 2016).

The pathogenic molecular mechanism of α-Syn in PD is complicated and not yet fully elucidated. Several studies have suggested that increased expression of α-Syn produces mitochondrial fragmentation, and this effect precedes any loss of mitochondrial function (Nakamura et al., 2011). Mitochondrial damage is closely related to α-Syn-induced PD pathogenesis, and an imbalance in mitochondrial fusion/fission dynamics leading to mitochondrial fragmentation is considered one factor that initiates mitochondrial damage in PD (Choi et al., 2006). Importantly, amino acid metabolism also acts as a critical signal to regulate mitochondrial fusion (Wai and Langer, 2016). Thus, the amino acid metabolism of neurons and the regulation of amino acid metabolism to promote autophagic α-Syn degradation and inhibit mitochondrial fragmentation in PD should be investigated.

To date, a few drugs that regulate amino acid metabolism have been developed, and some drugs are being evaluated in clinical trials or even widely used in clinical practice. L-asparaginase (L-ASNase), which was developed based on findings of amino acid deprivation, can catalyze the hydrolysis of asparagine and glutamine to aspartic acid and glutamate (Liao et al., 2017). As a therapeutic agent initially developed for acute lymphoblastic leukemia (Avramis and Tiwari, 2006), L-ASNase has been approved by the Food and Drug Administration (FDA). Environments deficient in asparagine and glutamine environments due to L-ASNase were shown to induce cell autophagy, and this effect is being widely studied for developing applications in other fields (Zhang et al., 2016). Moreover, a glutamine-deficient environment caused by L-ASNase also promotes mitochondrial fusion (Wai and Langer, 2016). Based on its ability to regulate amino acid metabolism, L-ASNase is a potential and promising approach for PD treatment.

In this study, we explored the potential effect of L-ASNase on SH-SY5Y cells overexpressing A53T mutant α-Syn (SH-SY5Y-A53T cells); these cells are considered a PD model that simulates pathological α-Syn aggregation, impairing normal mitochondrial dynamics (Xie and Chung, 2012). We found that L-ASNase administration activated the autophagic degradation of α-Syn in the SH-SY5Y-A53T cells without cytotoxicity at specific concentrations and times. Then, we determined the mechanism underlying L-ASNase—induced neuroprotection. We investigated abnormal amino acid metabolism in the cell model of PD and demonstrated that L-ASNase could function by targeting glutamine metabolism in PD treatment. Furthermore, autophagic degradation of α-Syn and mitochondrial fusion/fission dynamics were involved in L-ASNase—induced neuroprotection. Therefore, this study provides new data showing that L-ASNase could be a promising approach for PD treatment.

## Materials and Methods

### Establishment of SH-SY5Y-A53T Cells and Cell Culture

The SH-SY5Y cell line was purchased from the American type culture collection (ATCC, CRL-2266, USA), a Short Tandem Repeat (STR) authentication was measured to determine its identity before this experiment. The cells were stably overexpressing SNCA (A53T) by lentiviral particles. The sequence was synthesized according to Michael and colleagues (Polymeropoulos et al., 1997). The Genbank ID of wild-type (WT) α-Syn in the National Center for Biotechnology Information is L08850. In the A53T mutant α-Syn gene, the nucleotide at position 209 exerted a G → A missense mutation, which changed the alanine (Ala) at position 53 to threonine (Thr). Cells were cultured in Eagle’s Minimum Essential Medium (EMEM; GIBCO, Gaithersburg, MD, USA) containing 10% fetal bovine serum (FBS; GIBCO, Australia).

### Administration of Drugs

The L-ASNase was purchased from a commercial reagent company (MedChemExpress, HY-P1923, USA), performed in the form of lyophilized powder, stored at −20°C, dissolved in a buffered solvent (5 mM Tris-HCl 50 mM NaCl pH = 7.0) to a storage concentration of 500 IU/ml and stored at 4°C (not more than 2 weeks). 1-Methyl-4-phenylpy ridinium ion (MPP^+^; Sigam, D048, USA) was dissolved in dimethyl sulfoxide (DMSO; Thermo Fisher Scientific, 20688, USA) solution, with a storage concentration of 400 mM, and stored at −20°C in the dark. The drugs were dissolved in a fresh medium according to the working concentration, and the liquid of each experiment was newly prepared.

### Western Blot Analysis

Cells were lysed in RIPA buffer. Protein content was determined using Pierce BCA (Beyotime, China). Protein lysates were resolved in 10–15% sodium dodecyl sulfate-poly-acrylamide gel electrophoresis (SDS–PAGE), then transferred onto polyvinylidene fluoride (PVDF) membranes (Millipore, USA). Next, the membranes were reacted with primary antibodies overnight at 4°C, including rabbit anti-β-actin (Cell Signaling Technology, 4970), LC-3B (Cell Signaling Technology, 3868), SQSTM1/p62 (Abcam, 56416), α-synuclein (Cell Signaling Technology, 2642), Cytochrome C (Proteintech, 10993), Bax (Abcam 32503), Bcl2 (Abcam, 32124), Caspase3 (Cell Signaling Technology, 9662) and mouse anti-Caspase9 (Cell Signaling Technology, 9508). Subsequently, the membranes were incubated HRP-conjugated secondary antibodies, including goat anti-rabbit HRP 1:2,000 (Cell Signaling Technology, 7074) and goat anti-mouse HRP 1:2,000 (Cell Signaling Technology, 7076) at room temperature for 1 h. Finally, the membranes were detected by enhanced chemiluminescence detection reagent (ECL), the bands were captured with the Bio-Rad ChemiDoc MP system, and quantified with ImageJ software.

### Regulation and Detection of Autophagy

Ten micromolar chloroquine (CQ; Sigam, C6628, USA) for 24 h was used to block the autophagy process activated by L-ASNase, the use concentration/time referred to a previous report (Zeng et al., 2015). Autophagosomes were marked by cyto-ID (Enzo, 51031, USA) and lysosomes were marked by LysoTracker (Invitrogen, L12492, Waltham, MA, USA).

### Cell Viability Assay

Cell viability was detected by Cell Counting kit 8 (CCK-8; DojinDo, CK04, Japan), cells were seeded in a 96-well plate (Corning, USA) with at least 1 × 10^4^ cells per well, and 10 μl of reagent was added to each well. Next, this plate was incubated for 4 h, then the absorbance at 450 nm was detected using a Varioskan LUX Microplate Reader (Thermo Fisher Scientific, Waltham, MA, USA).

### Measurement of Mitochondrial Membrane Potential (MMP)

Cells were seeded in a confocal dish (Nest, China), a Jc-1 kit (Beyotime, C2005, China) was used to determine MPP, the JC-1 working solution was added to cell cultures at 37°C for 20 min, washed it twice quickly, and gently with JC-1 buffer before adding the complete medium. JC-1 monomers (λex 514 nm, λem 529 nm) and aggregates (λex 585 nm, λem 590 nm) were detected by a fluorescence confocal microscope (Leica Microsystems, Wetzlar, Germany) within 30 min. The relative ratio of red/green fluorescence was used as an indicator of MPP.

### Measurement of Intracellular ATP

Cells were seeded in a six-well plate, an ATP detection kit (Beyotime, S0026 China) was used to determine intracellular ATP contents. Two-hundred microliter lysate was added to each well, then pipetted it repeatedly. After the lysate was collected, the cells were centrifuged at 12,000 *g* at 4°C for 5 min, and the supernatant was taken for subsequent determination. According to the kit instructions, a standard curve was conducted, ATP working solution and samples were mixed in an opaque 96-well plate (Thomson, USA). The ATP concentration was calculated based on the RLU value measured in a luminescent plate (Thermo Fisher Scientific, Waltham, MA, USA). The protein concentration of each sample was determined using a BCA kit (Beyotime, China), and the final ATP concentration was converted into nmol/mg protein.

### Apoptosis Assay

Cell apoptosis was evaluated by an Annexin V-FITC/propidine iodide (PI) Kit (DojinDo, AD10, Japan). After treatment, cells were collected and washed by PBS, then re-suspended in binding buffer at a density of 1 × 10^6^ cells/ml. Next, the cells were reacted with Annexin V-FITC/PI reagent for 15 min in dark at 37°C, then analyzed by fluorescence-activated cell sorting using a Calibur flow cytometer (Becton-Dickinson, Franklin Lakes, NJ, USA).

### Immunofluorescence for Cleaved Caspase 3 and TUNEL Assay

Cells were seeded in a confocal dish (Nest, China). After treatment, cells were fixated with 4% paraformaldehyde for 30 min, then permeabilized with 0.3% Triton X-100 for 15 min, blocked with 10% normal goat serum (Solable, China) for 1 h, incubated with cleaved caspase 3 (1:400, Cell Signaling Technology, 9664) at 4°C overnight. Next, Cells were washed with PBS and incubated with a fluorescent secondary antibody (1:1,000, goat anti-Rabbit IgG H&L Cy3, Abcam 6939) for 1 h at room temperature. Then the cells were stained with terminal deoxynucleotidyl transferase (TdT) dUTP nick-end labeling (TUNEL) according to the manufacturer’s instructions of a TUNEL kit (Beyotime, C1086, China). Finally, nuclei were counterstained with DAPI.

### Sample Preparation for GC-MS/MS Targeted Amino Acid Metabolomics Analysis and GC-MS/MS Analysis

Cell collection and sample detection were referred to Ju et al. (2020). Shanghai Lu-Ming Biotech Company Limited (Shanghai, China) provided an experimental platform and assistance for the targeting amino acid metabolomics analysis. Briefly, a mixture of methanol/water (4:1 by volume) was used to collect 2 × 10^7^ per sample. Stored the sample in liquid nitrogen quickly. Before testing on the machine, equilibrated the sample to ambient temperature for 30 min, dispersed the sample by ultrasonic lysis method, concentrated and centrifuged, then freeze-dried. Finally, a mixture of BSTFA and n-hexane (4:1 by volume) was added to the sample and vortexed vigorously for 2 min, and derivatized at 70°C for 60 min. These samples were analyzed by a gas chromatography system (Thermo Fisher Scientific TSQ 9000, USA). UPLC-ESI-MS/MS was utilized as the analytical method for the quantitative detection of targeted amino acid metabolites.

### Intracellular Glutamine Content and Glutamine Synthetase (GS) Activity Measurement

A human glutamine ELISA assay kit was performed to detect intracellular glutamine content (Mlbio, ml064265, China). Glutamine Synthetase (GS) was measured according to Li et al. (2018) using GS test kits (Solable, BC0915, China).

### Mitochondrial Staining

To observe mitochondrial morphological changes, a Mito-Tracker Red kit (Invitrogen, M22425, USA) was used to label mitochondria. The images were randomized, cells were live imaged, and selected based on red fluorescence. The morphology of mitochondria was classified as a fragmented, tubular, or intermediate state in transfecting.

### Statistical Analysis

All data were analyzed by Graphpad Prism 7 software and expressed as mean ± SD. Differences between two means and among multiple means were assessed by unpaired two-tailed Student’s *t*-test. A comparison between multiple groups was analyzed through one-way ANOVA, *P* < 0.05 was considered a statistically significant difference.

## Results

### L-ASNase Administration at Specific Concentrations/Times Activated Autophagic Degradation of α-Syn in the SH-SY5Y-A53T Cell Model of PD Without Cytotoxicity

**Figure 1:**
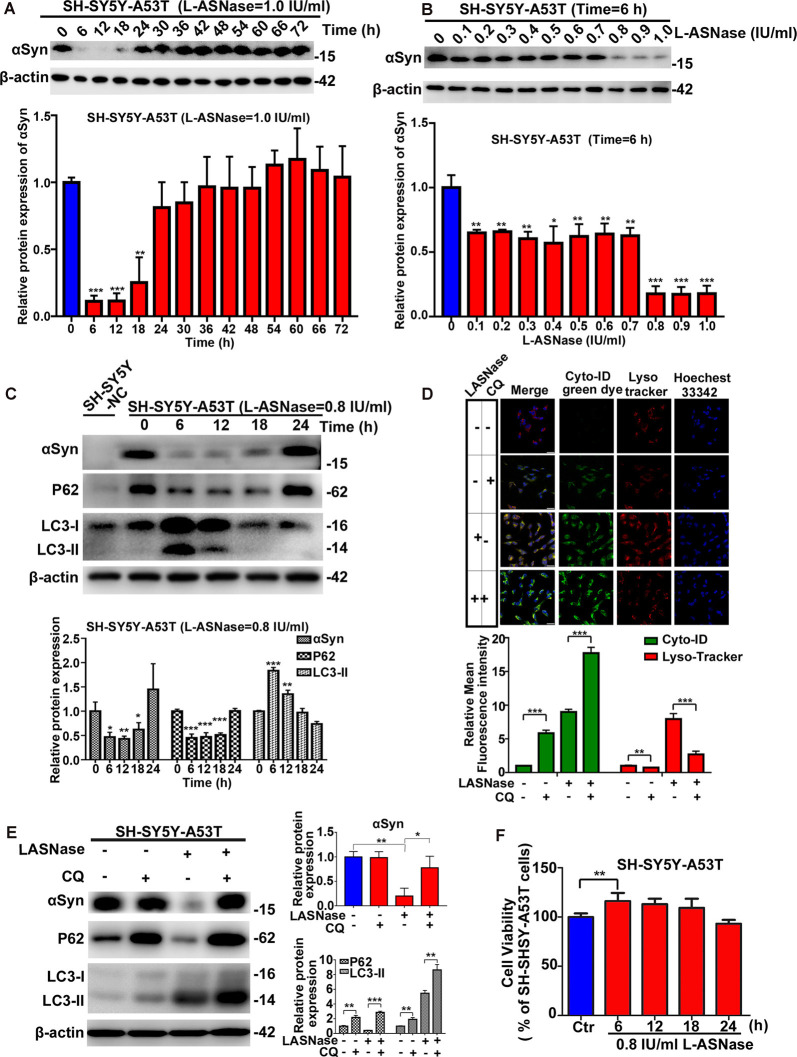
Effect of L-asparaginase (L-ASNase) on alpha-synuclein (α-Syn) autophagic degradation and cell viability in the SH-SY5Y-A53T Parkinson’s disease (PD) cell model. Western bolt results revealed that “0.8 IU/ml for 6 h” could be set as the specific concentration/time of L-ASNase to activated α-Syn autophagic degradation in SH-SY5Y-A53T cells step by step: **(A)** representative western bolt image indicating the effect of α-Syn autophagic degradation by 1 IU/ml L-ASNase from 0 to 72 h (the time point was set every 6 h), histogram reflecting the statistics and normalized to SH-SY5Y-A53T cells without L-ASNase treated (control), **P < 0.01, ***P < 0.001, **(B)** representative western bolt image showing the change of α-Syn accumulation induced by 0.1–1 IU/ml L-ASNase for 6 h, histogram reflecting the statistics and normalized to control, *P < 0.05, **P < 0.01, ***P < 0.001, **(C)** representative western bolt image showing the change of α-Syn accumulation, LC3-II and P62 caused by 0.8I U/ml from 0 to 24 h (the time point was set every 6 h), the protein sample in the first lane is from SH-SY5Y cells transfected with a negative control plasmid (marked as “SH-SY5Y NC”), histogram showing the relative protein expression of α-Syn, P62 and LC3-II in each group vs. “SH-SY5Y-A53T cells without L-ASNase treated”, *P < 0.05, **P < 0.01, ***P < 0.001. **(D)** Cells were stained with Cyto-ID and LysoTracker Red, confocal micrographs were taken at ×40, the bar was 10 μm, histogram reflecting the statistics of relative mean fluorescence intensity, **P < 0.01, ***P < 0.001. **(E)** Representative western bolt image showing the change of α-Syn accumulation, LC3-II, and P62 in each group, histogram showing the relative protein expression of α-Syn, P62, and LC3-II in each group vs. control, *P < 0.05, **P < 0.01, ***P < 0.001. **(F)** Histogram showing cell viability of SH-SY5Y-A53T cells with 0.8 IU/ml L-ASNase treated from 0 to 24 h (the time point was set every 6 h), statistical significance was presented as **P < 0.01. All the above statistics have been repeated three independent experiments, means ± SD.

First, we determined whether a suitable dose/time of L-ASNase administration without significant toxicity could effectively clear the excessive accumulation of the α-Syn protein in SH-SY5Y A53T cells (studied as the PD cell model). We explored the effect of different L-ASNase concentrations ranging from 1 to 4 IU/ml for 12 h. As shown in Supplementary Figures 1A,B, L-ASNase at 1 IU for 12 h significantly reduced the α-Syn accumulation in the SH-SY5Y A53T cells, increased the expression of LC3-II, and reduced P62 expression. Then, we tested the effect of 0–72 h (a time point was set every 6 h) of treatment at 1 IU/ml L-ASNase and found that the clearance at the 6 h time point was optimal (Figure 1A). To determine a minimum concentration/time of L-ASNase for α-Syn clearance, we tested the effect of 0.1–1 IU/ml L-ASNase for 6 h and found that the inhibitory effect was stably presented and highly significant at 0.8 IU/ml (Figure 1B). Thus, we used 0.8 IU/ml for 6 h as the specific concentration/time of L-ASNase treatment on SH-SY5Y A53T cells for further study. It was shown that L-ASNase administration at this specific concentration/time significantly decreased the α-Syn levels and induced autophagy in the SH-SY5Y A53T cell model (Figure 1C). To further verify whether L-ASNase promoted α-Syn degradation *via* autophagy, CQ was used to block autophagy flux. Pre-treatment with 10 μM CQ for 24 h could effectively block the autophagy process activated by L-ASNase, it caused the accumulation of autophagosomes, and then the combination of autophagosomes (marked by cyto-ID green dye) and lysosomes (marked by LysoTracker) was greatly reduced (Figure 1D). Western blot analysis also revealed the accumulation of autophagy marker proteins LC3-II and P62 induced by CQ (Figure 1E). After CQ blocked the autophagy flux, the clearance effect of α-Syn caused by L-ASNase was abolished (Figure 1E). Supplementary Figure 1C shows that no obvious morphological changes were detected in this PD model after the L-ASNase treatment. Figure 1F demonstrates that the cell viability of this PD model with L-ASNase administration at the specific concentration/time was higher than that of the cells without L-ASNase treatment. However, as the administration time of L-ASNase prolonged, it would damage the cells (Supplementary Figure 1D).

### L-ASNase Improved Mitochondrial Function in the SH-SY5Y-A53T Model, and L-ASNase Pretreatment Reduced the Mitochondrial Damage Caused by MPP^+^ Exposure in These Cells

**Figure 2:**
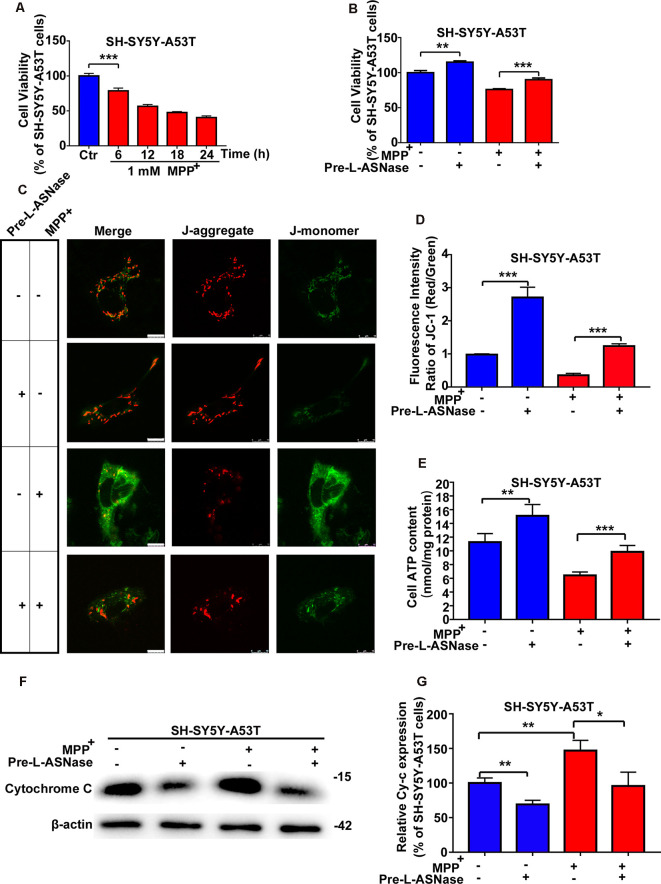
Effect of L-ASNase on mitochondrial function of the SH-SY5Y-A53T PD cell model with or without MPP^+^ exposed. **(A)** Histogram showing cell viability of SH-SY5Y-A53T cells with 1 mM MPP^+^ exposed from 0 to 24 h (the time point was set every 6 h). **(B–G)** Each group represented cells with different processing methods, as shown below, the first group represented SH-SY5Y-A53T cells, the second group represented SH-SY5Y-A53T cells with 0.8 IU/ml L-ASNase treated for 6 h, the third group represented SH-SY5Y-A53T cells with 1 mM MPP^+^ exposed for 6 h, the fourth group represented SH-SY5Y-A53T cells with L-ASNase pretreated and MPP^+^ exposed: **(B)** histogram showing the cell viability of each group, **(C)** representative JC-1 staining fluorescence images taken from a laser confocal microscope of each group, the bar was 10 μm, **(D)** histogram showing the fluorescence intensity ratio of Red/Green of each group, **(E)** histogram showing the intracellular ATP levels (nmol/mg protein) of each group, **(F)** representative western bolt analysis of Cytochrome C (Cy-c) expression in cytoplasm of each group, and **(G)** histogram showing the relative Cy-c expression (represented as % of SH-SY5Y-A53T cells) of each group, the data were presented as means ± SD of three independent experiments. Statistical significance was described as *P < 0.05, **P < 0.01, ***P < 0.001.

The pathogenesis of PD has been reported to be attributed to gene-environment interactions, and the A53T α-Syn mutation is a common genetic variant that increases susceptibility to MPP^+^, leading to oxidative stress and mitochondrial dysfunction (Zhang et al., 2017). Consistent with previous reports (Ma et al., 2010), compared to the WT cells, the SH-SY5Y-A53T cells exhibited a more pronounced decrease in cell viability after exposure to MPP^+^. In line with our previous study (Li et al., 2019), impairment in cell viability became remarkable after 24 h after 1 mM MPP+ treatment in WT SH-SY5Y cells (data was repeated in Supplementary Figure 2A). In this study, the decline in cell viability became significant after just 6 h in SH-SY5Y-A53T cells (Figure 2A). In this study, exposure to 1 mM MPP^+^ for 6 h was used as the environmental risk factor. As shown in Figure 2B, L-ASNase treatment improved the viability of the SH-SY5Y-A53T cells (Figure 2B; **P < 0.01, Group 2 vs. 1), and pretreatment of L-ASNase could improve the cell viability of SH-SY5Y-A53T cells exposed to MPP^+^ (Figure 2B, ***P < 0.001, Group 4 vs. 3). However, compared with the improvement of cell viability of SH-SY5Y-A53T by L-ASNase, the improvement of cell viability of WT SH-SY5Y by L-ASNase was not significant (Supplementary Figure 2B). It was reported that A53T α-Syn mutation caused severe mitochondrial damage in SH-SY5Y cells (Butler et al., 2012), which was also be confirmed in our study (Supplementary Figure 2C). To determine whether L-ASNase can improve mitochondrial function in the SH-SY5Y-A53T model and reduce the mitochondrial damage of those cells caused by MPP^+^, we assessed relevant indicators including MMP change, ATP formation, and cytochrome C release. Representative JC-1 staining fluorescence images were displayed in Figure 2C. MMP was assessed by JC-1 staining, and the fluorescence intensity ratio of red/green was distinctly higher in the L-ASNase treated/pretreated groups than the control groups (Figure 2D, ***P < 0.001, Group 2 vs. 1; ***P < 0.001, Group 4 vs. 3). Intracellular ATP levels were detected using an assay kit, ATP content was expressed as nmol/mg protein, and the value increased notably in the L-ASNase-treated/pretreated groups (Figure 2E, **P < 0.01, Group 2 vs. 1; ***P < 0.001, Group 4 vs. 3). Cytochrome C release was analyzed by western blots for cytoplasmic protein, as shown in Figure 2F. L-ASNase treatment/pretreatment decreased the expression of cytochrome C in the SH-SY5Y-A53T model, and these cells were exposed to MPP^+^; the statistical data are shown in Figure 2G (**P < 0.01, Group 2 vs. 1; *P < 0.05, Group 4 vs. 3). These results demonstrated the neuroprotective effect of L-ASNase functioned *via* improving mitochondrial function in a cell model of PD.

### L-ASNase Pretreatment Relieved Apoptosis in the Mitochondrial Pathway of the SH-SY5Y-A53T Model After MPP^+^ Exposure

**Figure 3:**
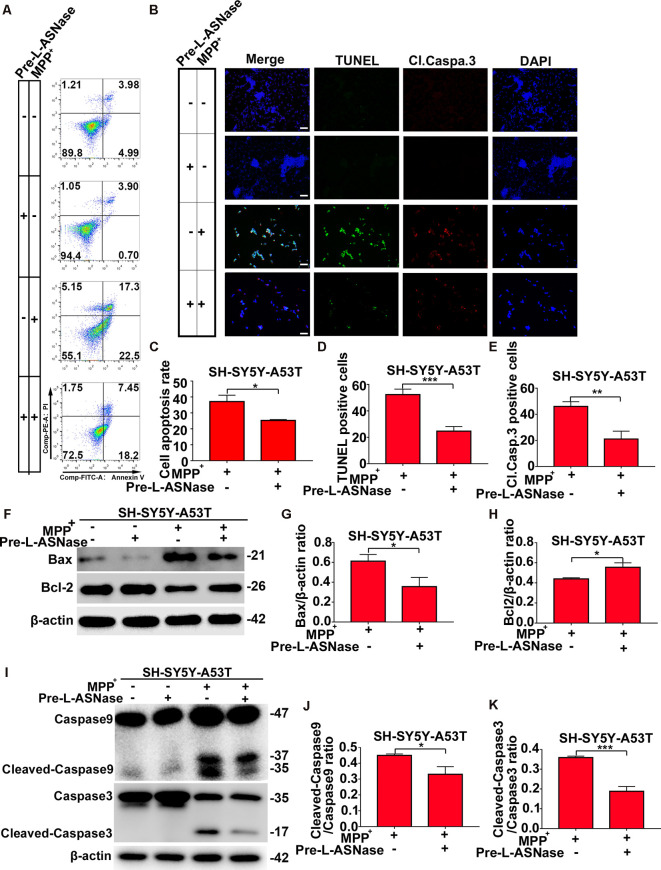
Effect of L-ASNase on apoptosis in the mitochondrial pathway in the SH-SY5Y-A53T PD cell model with MPP^+^ exposure. **(A)** Representative flow cytometry result of cells in each group stained with Annexin V/PI. **(B)** Representative images of TUNEL assays, cleaved-caspase 3, and DAPI immunostaining of each group, the bar was 50 μm. **(C)** Histogram showing the cell apoptosis measured by Annexin V/PI staining in SH-SY5Y-A53T cells to MPP^+^ exposure with or without L-ASNase pretreated, *P < 0.05. **(D)** Histogram showing the ratio of TUNEL positive cells in SH-SY5Y-A53T cells exposed to MPP^+^ with or without L-ASNase pretreatment, ***P < 0.001. **(E)** Histogram showing the ratio of cleaved-caspase 3 positive cells in these two groups, **P < 0.01. **(F)** Representative western bolt analysis of Bax and Bcl2 expression in each group. **(G)** Histogram showing the ratio of Bax/β-actin protein expression levels in SH-SY5Y-A53T cells exposed to MPP^+^ with or without L-ASNase pretreatment, *P < 0.05. **(H)** Histogram showing the ratio of Bcl2/β-actin protein expression levels in these two groups, *P < 0.05. **(I)** Representative western bolt analysis of cleaved caspase-9, caspase-9, cleaved caspase-3, and caspase-3 expression in each group. **(J)** Histogram showing the ratio of cleaved caspase-9/caspase-9 protein expression levels in SH-SY5Y-A53T cells exposed to MPP^+^ with or without L-ASNase pretreatment, *P < 0.05. **(K)** Histogram showing the ratio of cleaved caspase-3/caspase-3 protein expression levels in these two groups, ***P < 0.001. The data were presented as means ± SD of three independent experiments.

We found that overexpressing the A53T α-Syn gene in SH-SY5Y neuroblastoma cells did not significantly trigger apoptosis, but apoptosis was increased significantly when SH-SY5Y-A53T cells were exposed to MPP^+^ (data not shown), which was consistent with the results of previous studies (Ma et al., 2010). Then, we tested whether pretreatment with L-ASNase could effectively reduce the apoptosis of the SH-SY5Y-A53T cells after MPP^+^ exposure. As shown in Figures 3A,C, cell apoptosis was analyzed by fluorescence-activated cell sorting, and L-ASNase pretreatment reduced the apoptosis rate of the SH-SY5Y-A53T cells exposed to MPP^+^ (*P < 0.05). To further verify the protective effect of L-ASNase against the MPP^+^-induced apoptosis in the SH-SY5Y-A53T cells, we performed TUNEL assays and assessed cleaved caspase-3 *via* electron microscopy (Figure 3B). L-ASNase pretreatment reduced the percentages of both the TUNEL-positive cells (Figure 3D, ***P < 0.001) and the cleaved caspase 3 positive cells (Figure 3E, **P < 0.01), which further confirmed this protective effect of L-ASNase. As mentioned above, L-ASNase pretreatment can protect mitochondrial function in the PD model, and indicators of the mitochondrial apoptotic pathway, including Bax, Bcl2, caspase-9, and caspase-3 cleavage, were examined by western blot analyses (Figures 3F–K). The results revealed that the expression of Bax, cleaved caspase-9/caspase-9, and cleaved caspase-3/caspase-3 could be reduced with L-ASNase pretreatment in the cell model of PD (Figure 3G, *P < 0.05; Figure 3J, *P < 0.05; Figure 3K, ***P < 0.001), while the expression of Bcl2 was increased (Figure 3H, *P < 0.05). These results revealed the neuroprotective effect of L-ASNase was involved in the inhibition of apoptosis in the mitochondrial pathway of a PD model.

### The Apoptosis Reduced by L-ASNase in the SH-SY5Y-A53T Model With MPP^+^ Exposure Could be Significantly Restored by CQ

**Figure 4:**
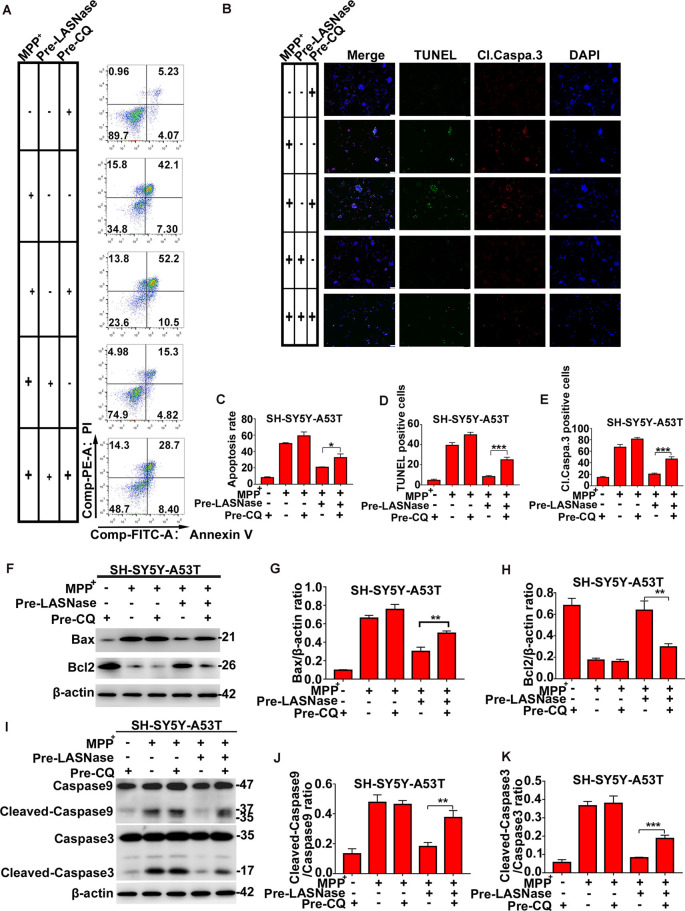
The decreased apoptosis by L-ASNase in the SH-SY5Y-A53T model with MPP^+^ exposure was raised by chloroquine (CQ). **(A)** Representative flow cytometry result of cells in each group stained with Annexin V/PI. **(B)** Representative images of TUNEL assays, cleaved-caspase 3, and DAPI immunostaining of each group, the bar was 50 μm. **(C)** Histogram showing the cell apoptosis measured by Annexin V/PI staining in each group, *P < 0.05. **(D)** Histogram showing the ratio of TUNEL positive cells in each group, ***P < 0.001. **(E)** Histogram showing the ratio of cleaved-caspase 3 positive cells in each group, **P < 0.01. **(F)** Representative western bolt analysis of Bax and Bcl2 expression in each group. **(G)** Histogram showing the ratio of Bax/β-actin protein expression levels in each group, **P < 0.01. **(H)** Histogram showing the ratio of Bcl2/β-actin protein expression levels in each group, **P < 0.01. **(I)** Representative western bolt analysis of cleaved caspase-9, caspase-9, cleaved caspase-3, and caspase-3 expression in each group. **(J)** Histogram showing the ratio of cleaved caspase-9/caspase-9 protein expression levels in each group, **P < 0.01. **(K)** Histogram showing the ratio of cleaved caspase-3/caspase-3 protein expression levels in each group, ***P < 0.001. The data were presented as means ± SD of three independent experiments.

To fully study the relationship between the decreased apoptosis induced by L-ASNase in PD cell models and L-ASNase induced autophagy, CQ was used to block autophagy. As shown in Figures 4A,B, cell apoptosis was analyzed by fluorescence-activated cell sorting and TUNEL/cleaved caspase 3 stainings. When the SH-SY5Y-A53T cells were co-pretreated with L-ASNase and CQ (cells were first pretreated with CQ for 24 h, and then pretreated with L-ASNase for 6 h), the apoptosis triggered by MPP^+^ was increased compared to that pretreated with L-ASNase alone (Figures 4C–E, *P < 0.05, ***P < 0.001 Group 5 vs. 4). Further, we analyzed the expression of Bax, Bcl2, caspase-9, and caspase-3 cleavage (Figures 4F–K). Compare to that pretreated with L-ASNase alone, the ratios of Bax/β-actin, cleaved caspase-9/caspase-9, and cleaved caspase-3/caspase-3 were increased when co-pretreated with L-ASNase and CQ (Figure 4G, **P < 0.01 Group 5 vs. 4; Figure 4J, **P < 0.01 Group 5 vs. 4; Figure 4K, Group 5 vs. 4 ***P < 0.001), while the Bcl2/β-actin ratio was reduced (Figure 4H, **P < 0.01 Group 5 vs. 4). These results suggested that the decreased apoptosis by L-ASNase was mediated by autophagy at least to some extent.

### GC-MS/MS Targeted Amino Acid Metabolomics Analysis of the Control, the SH-SY5Y-A53T Cell Model of PD, and the L-ASNase-Treated Cell Model of PD

**Figure 5:**
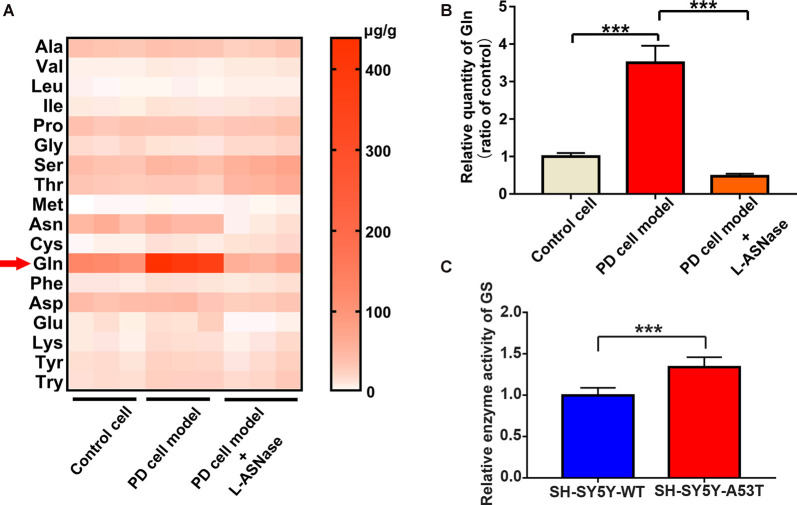
GC-MS/MS targeted amino acid metabolisms analysis of control, SH-SY5Y-A53T PD cell model and L-ASNase treated PD cell model. **(A,B)** Each group represented cells with different processing methods, as shown below, the first group represented the control cells (SH-SY5Y neuroblastoma cells), the second group represented the PD cell model (SH-SY5Y-A53T cells), the third group represented the PD cell model with L-ASNase treatment: **(A)** statistical heat map showing 18 kinds of human essential amino acids in these three groups, including Alanine (Ala), Valine (Val), Leucine (Leu), Isoleucine (Ile), Dl-proline (Pro), Glycine (Gly), Serine (Ser), Threonine (Thr), Methionine (Met), Asparagine (Asn), Cysteine (Cys), Glutamine (Gln), Phenylalanine (Phe), Aspartic acid (Asp), Glutamic acid (Glu), Lysine (Lys), Tyrosine (Tyr) and Tryptophan (Try), samples were derived from three independent experiments; **(B)** histogram showing the relative quantity of intracellular glutamine in these three groups, normalized to control cells, ***P < 0.001. **(C)** Histogram showing the relative enzyme activity of Glutamine Synthetase (GS), normalized to wild-type (WT) SH-SY5T cells, ***P < 0.001. All the above data were presented as means ± SD of three independent.

As mentioned above, L-ASNase could regulate amino acid metabolism and had a protective effect on the PD model. To further determine the related mechanisms, we detected of the levels of intracellular amino acids by GC-MS/MS targeted amino acid metabolisms analysis in a control cell model (SH-SY5Y neuroblastoma cells), the PD model (A53T was transiently overexpressed in the SH-SY5Y cells), and the PD model treated with L-ASNase, respectively. Except for arginine and histidine, 18 kinds of essential amino acids in humans were detected (arginine is converted into ornithine during derivatization, which cannot be accurately separated qualitatively and quantitatively; histidine is not stable in the GC-MS/MS system and thus was not included in this analysis). The related heat map is given in Figure 5A. It revealed that glutamine metabolism had the most significant difference between each group. To further verify this result, the quantitative determination by a glutamine ELISA assay kit was performed, compared with that of the control cells, glutamine dramatically increased in the PD cell model. Notably, the high level of glutamine could be significantly reduced after L-ASNase treatment in the PD cell model (Figure 5B, ***P < 0.001, Group 2 vs. 1; ***P < 0.001). Then, the activity of glutamine synthetase (GS) was examined by GS test kits. Compared with WT SH-SY5Y cells, the activity of GS was increased in SH-SY5Y-A53T cells (Figure 5C). High activity of GS might be responsible for the increased content of glutamine in SH-SY5Y-A53T cells. Taken together, these results indicated that glutamine metabolism was the potential target for L-ASNase in PD treatment.

### Glutamine Supplementation Reduced the Neuroprotective Effect of L-ASNase on the SH-SY5Y-A53T Cells

**Figure 6:**
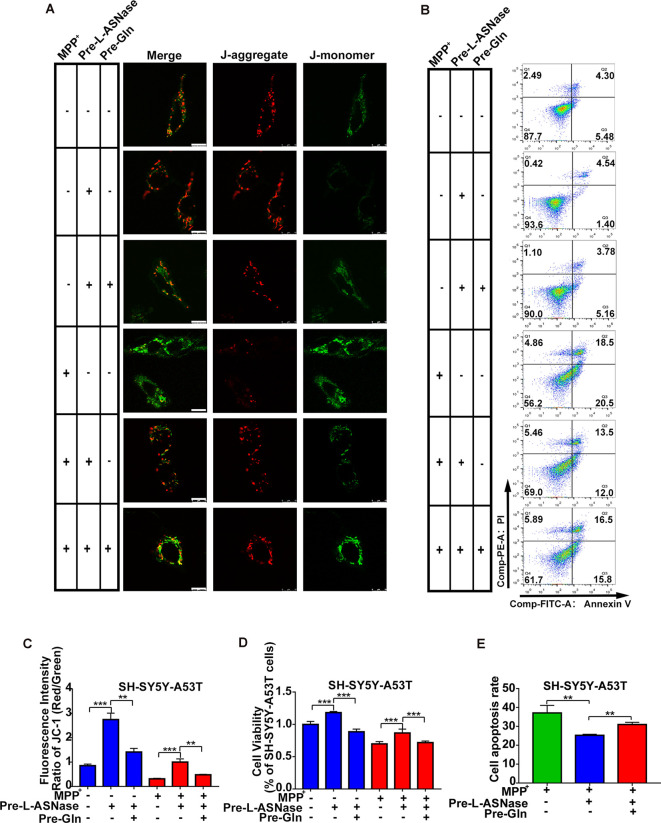
Effect of glutamine supplementation on L-ASNase induced neuroprotection in the SH-SY5Y-A53T PD cell model. **(A–D)** Each group represented cells with different processing methods, as shown below, the first group represented SH-SY5Y-A53T cells, the second group represented SH-SY5Y-A53T cells with L-ASNase treatment, the third group represented SH-SY5Y-A53T cells with L-ASNase and glutamine pretreated, the fourth group represented SH-SY5Y-A53T cells with MPP^+^ exposed, the fifth group represented SH-SY5Y-A53T cells with L-ASNase pretreated before exposure to MPP^+^, the sixth group represented SH-SY5Y-A53T cells with L-ASNase and glutamine pretreated before exposure to MPP^+^, glutamine was abbreviated as Gln: **(A)** representative JC-1 staining fluorescence images of each group, the bar was 10 μm, **(B)** representative flow cytometry result of cells in each group stained with Annexin V/PI, **(C)** histogram showing the fluorescence intensity ratio of Red/Green of each group, **(D)** histogram showing the cell viability (represented as % of SH-SY5Y-A53T cells) of each group. **(E)** Histogram showing the cell apoptosis measured by Annexin V/PI staining in the groups exposed to MPP^+^. The data were described as means ± SD of three independent experiments, **P < 0.01, ***P < 0.001.

Next, we determined whether inhibition of L-ASNase -induced glutamine deprivation could weaken the neuroprotective effect of L-ASNase on the cell model of PD. We focused on the effects of L-ASNase and glutamine on the MMP, cell viability, and cell apoptosis. The dose of glutamine added during L-ASNase administration was according to Liao et al. (2017), and cell-free medium containing glutamine (30 mg/dl) was incubated with 0.8 IU/ml L-ASNase for 6 h before MPP^+^ exposure. As shown in Figures 6A,C, the fluorescence intensity ratio of red/green (JC-1 staining) was increased by L-ASNase treatment/pretreatment in the PD model with or without MPP^+^ exposed (Figure 6C, ***P < 0.001, Group 2 vs. 1; ***P < 0.001, Group 5 vs. 4), but the L-ASNase-mediated improvement in the MMP could be suppressed by simultaneous addition of glutamine (Figure 6C, **P < 0.01, Group 3 vs. 2; **P < 0.01, Group 6 vs. 5). Similarly, the cell viability could be improved by L-ASNase treatment/pretreatment in the PD model with or without MPP^+^ exposure (Figure 6D, ***P < 0.001, Group 2 vs. 1; ***P < 0.001, Group 5 vs. 4), and this effect could also be restrained by glutamine supplementation (Figure 6D, ***P < 0.001, Group 3 vs. 2; ***P < 0.001, Group 6 vs. 5). Furthermore, this treatment resulted in cell apoptosis of the PD model after exposure to MPP^+^. A representative flow cytometric result is shown in Figure 6B. Cell apoptosis was increased by L-ASNase pretreatment in the PD model with MPP^+^ exposure (Figure 6E, **P < 0.01, Group 2 vs. 1), but was reduced by simultaneous addition of glutamine (Figure 6E, **P < 0.01, Group 3 vs. 2).

### Glutamine Supplementation Reduced the L-ASNase-Induced Autophagic Degradation of α-Syn and Mitochondrial Fusion in the SH-SY5Y-A53T Model

**Figure 7:**
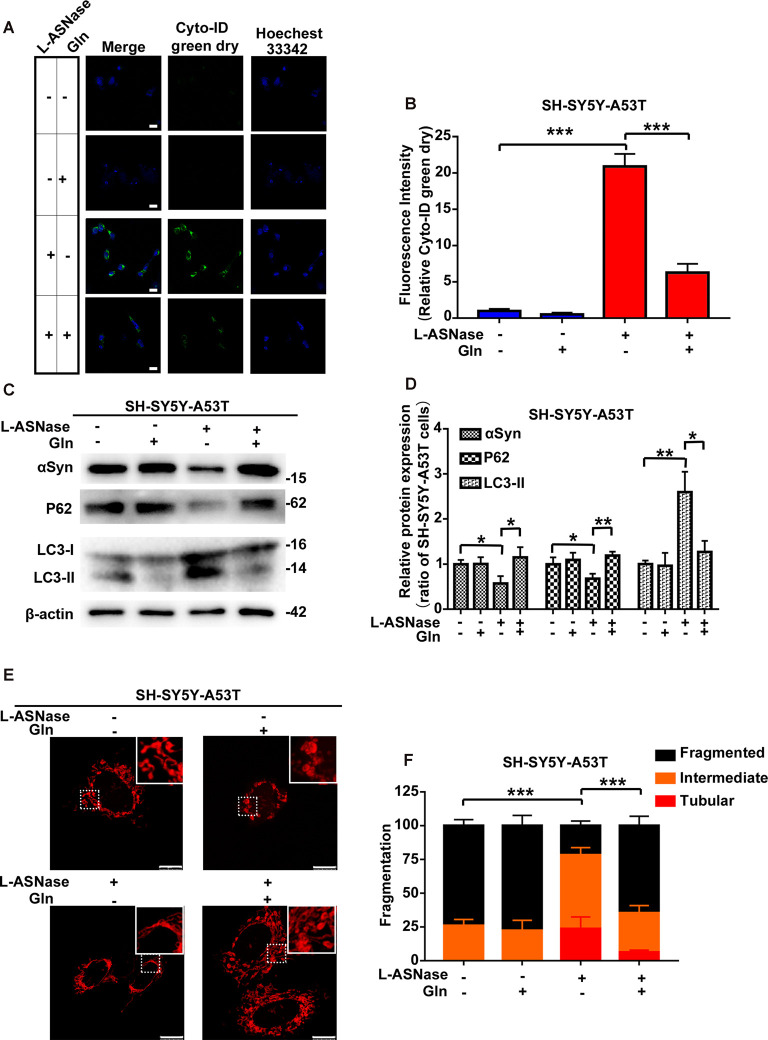
Effect of glutamine supplementation on L-ASNase induced α-Syn autophagic degradation and mitochondrial fusion in the SY5Y-A53T PD cell model. **(A–F)** Each group represented cells with different processing methods, as shown below, the first group represented SH-SY5Y-A53T cells, the second group represented SH-SY5Y-A53T cells with glutamine treatment, the third group represented SH-SY5Y-A53T cells with L-ASNase treatment, the fifth group represented SH-SY5Y-A53T cells with L-ASNase and glutamine treated simultaneously, glutamine was abbreviated as Gln: **(A)** representative images of Cyto-ID Green dye and Hoechest 33342 immunostaining of each group, the bar was 25 μm, **(B)** histogram showing the fluorescence intensity of relative Cyto-ID green dry (represented as % of SH-SY5Y-A53T cells) in each group, **(C)** representative western blot analysis of α-Syn accumulation and autophagy related protein expression (P62 and LC3) in each group, **(D)** histogram showing the relative α-Syn, P62 and LC3-II expression (represented as % of SH-SY5Y-A53T cells) in each group, **(E)** representative images of mitochondrial morphology stained by Mito-Tracker Red in each group, the bar was 10 μm, and **(F)** statistical graph showing the percentage of various morphological states in each group, mitochondrial morphology was classified as fragmented, tubular, or intermediate. The data were presented as means ± SD of three independent experiments. Statistical significance was presented as *P < 0.05, **P < 0.01, ***P < 0.001.

Our results showed that L-ASNase administration at a specific concentration/time had a neuroprotective effect on a cell model of PD. Analysis of amino acid metabolism showed that L-ASNase caused a certain degree of amino acid deprivation, and the regulation of glutamine metabolism may be the most critical target for L-ASNase. Deprivation of amino acids, including glutamine, was shown to activate autophagy, and glutamine deprivation was a key signal to promote mitochondrial fusion (Rambold et al., 2011; Wai and Langer, 2016). We further investigated the effects of L-ASNase on autophagic clearance of α-Syn, mitochondrial fusion, and the role of glutamine in this process. As shown in Figure 7A, Cyto-ID Green dye was applied to mark autophagic vesicles. Green (Cyto-ID) dots increased significantly with L-ASNase treatment in the PD model, but this activation could be significantly suppressed by the simultaneous addition of glutamine (30 mg/dl), and the relative Cyto-ID fluorescence intensity was calculated (Figure 7B, ***P < 0.001, Group 3 vs. 1; ***P < 0.001, Group 4 vs. 3). Then, the influence of this manipulation on α-Syn accumulation and autophagic protein changes (P62 and LC3) was examined, as shown in Figures 7C,D. The protein levels of α-Syn and P62 were decreased by L-ASNase treatment but increased by the simultaneous addition of glutamine (Figure 7D, α-Syn, *P < 0.05, Group 3 vs. 1, *P < 0.05, Group 4 vs. 3; P62, *P < 0.05, Group 3 vs. 1, **P < 0.01, Group 4 vs. 3). Conversely, the protein level of LC3-II increased with L-ASNase treatment but decreased with the simultaneous addition of glutamine (Figure 7D, LC3-II, **P < 0.01, Group 3 vs. 1, *P < 0.05, Group 4 vs. 3). The above results indicated that inhibition of L-ASNase-induced glutamine deprivation could reduce the L-ASNase-induced autophagic level and autophagic degradation of α-Syn in the SH-SY5Y-A53T cell model of PD. Moreover, our results demonstrated that the protective effect of L-ASNase on the PD model is mainly due to the improvement in mitochondrial function. Aggregated α-Syn directly interacted with the mitochondrial membrane to drive mitochondrial fission, which could eventually impair mitochondrial function and cause apoptosis (Choi et al., 2006; Nakamura et al., 2011). Owing to the ability of glutamine metabolism to regulate mitochondrial dynamics, we further focused on mitochondrial dynamics. As shown in Figures 7E,F, the Mito Tracker Red kit was used to assess mitochondrial morphology. Mitochondrial fragmentation was reduced significantly by L-ASNase treatment in the PD model, but this inhibitory effect of L-ASNase on mitochondrial fission disappeared with the simultaneous administration of glutamine (Figure 7F, ***P < 0.001, Group 3 vs. 1, ***P < 0.001, Group 4 vs. 3).

## Discussion

Previous studies have reported that L-ASNase showed a therapeutic effect on many diseases involving abnormal amino acid metabolism (Dhankhar et al., 2020). In this study, we first revealed that the glutamine level in a cell model of PD was significantly increased compared with that in control cells. The abnormal glutamine levels indicated that L-ASNase might be a potential approach for PD treatment. However, the efficiency and molecular mechanisms of L-ASNase-based therapy for PD have not been fully elucidated. Thus, we demonstrated for the first time that L-ASNase has a neuroprotective effect in a PD model *via* a moderate glutamine deprivation. Moreover, L-ASNase exhibited a strong neuroprotective effect through α-Syn degradation.

Given that glutamine is an important nutrient for cell metabolism, the doses of L-ASNase used in PD treatment must be rigorous without the side effect of excessive glutamine depletion. Further, with the action time of the L-ASNase extending, L-ASNase’s effect on autophagy activation and α-Syn degradation was diminished, which might result from excessive deprivation of essential amino acids caused by the exceeding activity of L-ASNase. In this study, we chose the minimum concentration at which L-ASNase induced autophagic degradation of α-Syn to conduct the subsequent experiments. This specific dose of L-ASNase, which induces a certain degree of glutamine deprivation, maybe a suitable one to exert a prominent neuroprotective effect in the cell model of PD.

To further understand the biological mechanisms of L-ASNase in PD therapy, we searched the published literature. A study showed that glutamine metabolism and autophagy constituted a specific metabolic program, and in this program, glutamine metabolism could regulate autophagic activity (Tan et al., 2017). Autophagy is a degradative pathway for aggregated or dysfunctional cellular proteins, and some studies have indicated that degrading pathogenesis-related proteins in PD through pharmacological autophagic activation could be an effective therapeutic approach (Hebron et al., 2013; Guo et al., 2016; Dahmene et al., 2017). In PD, the accumulation of misfolded oligomers and large aggregates of α-Syn exhibits neurotoxic effects and contributes to neurodegeneration (Bengoa-Vergniory et al., 2017; Froula et al., 2018). Thus, we hypothesized that L-ASNase activated autophagy to promote α-Syn degradation by regulating glutamine metabolism. To confirm this, we added glutamine to the cell model of PD after treatment with L-ASNase. Then, we found that the autophagic level increased and the autophagic degradation of α-Syn induced by L-ASNase was reversed. These data strongly indicated that the autophagic degradation of α-Syn is involved in the effects of L-ASNase on PD.

Moreover, for the full elucidation of the relationship between the degradation of α-Syn aggregates and neuron viability in PD, further studies are needed to detect the downstream targets impacted by α-Syn that lead to neurodegeneration. The expression of α-Syn was reported to cause the fragmentation of mitochondria in neurons (Nakamura et al., 2011), while many neurodegenerative diseases, including PD, exhibit abnormal mitochondrial morphology and biochemical dysfunction. Loss of the dynamic balance of mitochondrial fusion and fission resulted in neuronal death and accelerated neurodegeneration (Choi et al., 2006). In this study, our data indicated that the fragmentation of mitochondria resulted in mitochondrial fusion in a cell model of PD treated with L-ASNase when α-Syn was significantly degraded by activated autophagy. These results could account for the neuroprotective effect of L-ASNase in PD.

Low glutamine levels were reported to trigger substantial mitochondrial fusion by diluting damaged mitochondrial proteins and repairing damage to preserve the integrity of mitochondrial DNA (Rambold et al., 2011; Wai and Langer, 2016). A study from *Cell Research* showed that cellular glutaminase 1 (GLS1) could act as a mediator between glutamine deprivation and the fusion of mitochondria (Cai et al., 2018). However, little is known about the exact mechanism of accelerated mitochondrial fusion induced by glutamine deprivation. The regulatory relationship between glutamine and mitochondrial fusion remains unclear. In PD, our study demonstrated that appropriate deprivation of glutamine triggered autophagic degradation of α-Syn, and then, mitochondrial fusion was activated to maintain mitochondrial integrity. Thus, identification of whether L-ASNase triggered mitochondrial fusion *via* autophagic clearance of α-Syn alone or other pathways should be investigated in the future.

**Figure 8:**
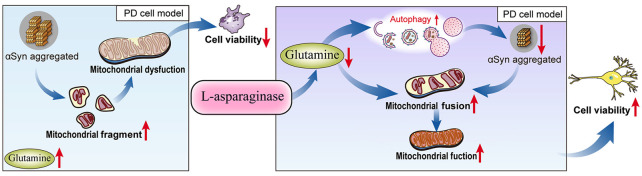
Schematic model for L-ASNase performing neuroprotective effect in PD cell model *via* regulating glutamine metabolism. The SH-SY5Y-A53T PD cell model mimicked pathological α-Syn aggregation, increasing mitochondrial fragments, accompanied by intracellular glutamine accumulation. L-ASNase treatment at the dose of inducing α-Syn autophagic degradation can cause proper glutamine deprivation, which leads to autophagy activation and direct or indirect mitochondrial fusion, thereby improving mitochondrial function and exerting neuroprotective effects.

Taken together, our results demonstrated for the first time that L-ASNase had a neuroprotective effect on a cell model of PD. After treatment with L-ASNase under specific conditions, cell mitochondrial function was improved, and mitochondrial apoptosis was rescued in the SH-SY5Y-A53T cells with or without MPP^+^ exposure. Notably, compared with those in the control cells, the glutamine level was significantly increased in the PD model, and L-ASNase treatment reduced the glutamine level in these cells. To further study the role of glutamine metabolism in L-ASNase treatment in the PD model, we simultaneously added glutamine and L-ASNase to the cell model of PD to inhibit L-ASNase -induced glutamine deprivation. The results showed that glutamine supplementation weakened the neuroprotective effect of L-ASNase on the PD model and reduced the L-ASNase-induced autophagic degradation of α-Syn and mitochondrial fusion. These results revealed that the intrinsic mechanism of L-ASNase treatment in PD is related to an appropriate deprivation of glutamine, which results in autophagic activation and direct or indirect mitochondrial fusion. The related mechanisms are shown in a schematic diagram (Figure 8). Since PD is a progressive disease with a long disease course, the suitable dose and time of L-ASNase *in vivo* should be carefully identified. The long-term safety of L-ASNase should be fully analyzed in animal models in future studies. In summary, we demonstrated that the targeted regulation of glutamine metabolism by L-ASNase administration could be a promising strategy for PD therapy.

## Data Availability Statement

All datasets presented in this study are included in the article/Supplementary Material.

## Ethics Statement

This article does not contain any studies with human participants or animals performed by any of the authors.

## Author Contributions

LJW, KN, and YZ designed the study. QZ, YG, and JZ performed the experiments. YL, JC, RH, and GM analyzed the experimental data. QZ, YG, and LMW wrote the manuscript. All authors read and approved the manuscript. All authors contributed to the article and approved the submitted version.

## Conflict of Interest

The authors declare that the research was conducted in the absence of any commercial or financial relationships that could be construed as a potential conflict of interest.
