# Supplementary material for: L-Asparaginase Exerts Neuroprotective Effects in an SH-SY5Y-A53T Model of Parkinson’s Disease by Regulating Glutamine Metabolism
Source: Front Mol Neurosci. 2020 Sep 30;13:563054. doi: 10.3389/fnmol.2020.563054 (PMC7557534; doi:10.3389/fnmol.2020.563054)
Supplement: Supplementary file 1 [file Data_Sheet_1.DOCX]

**
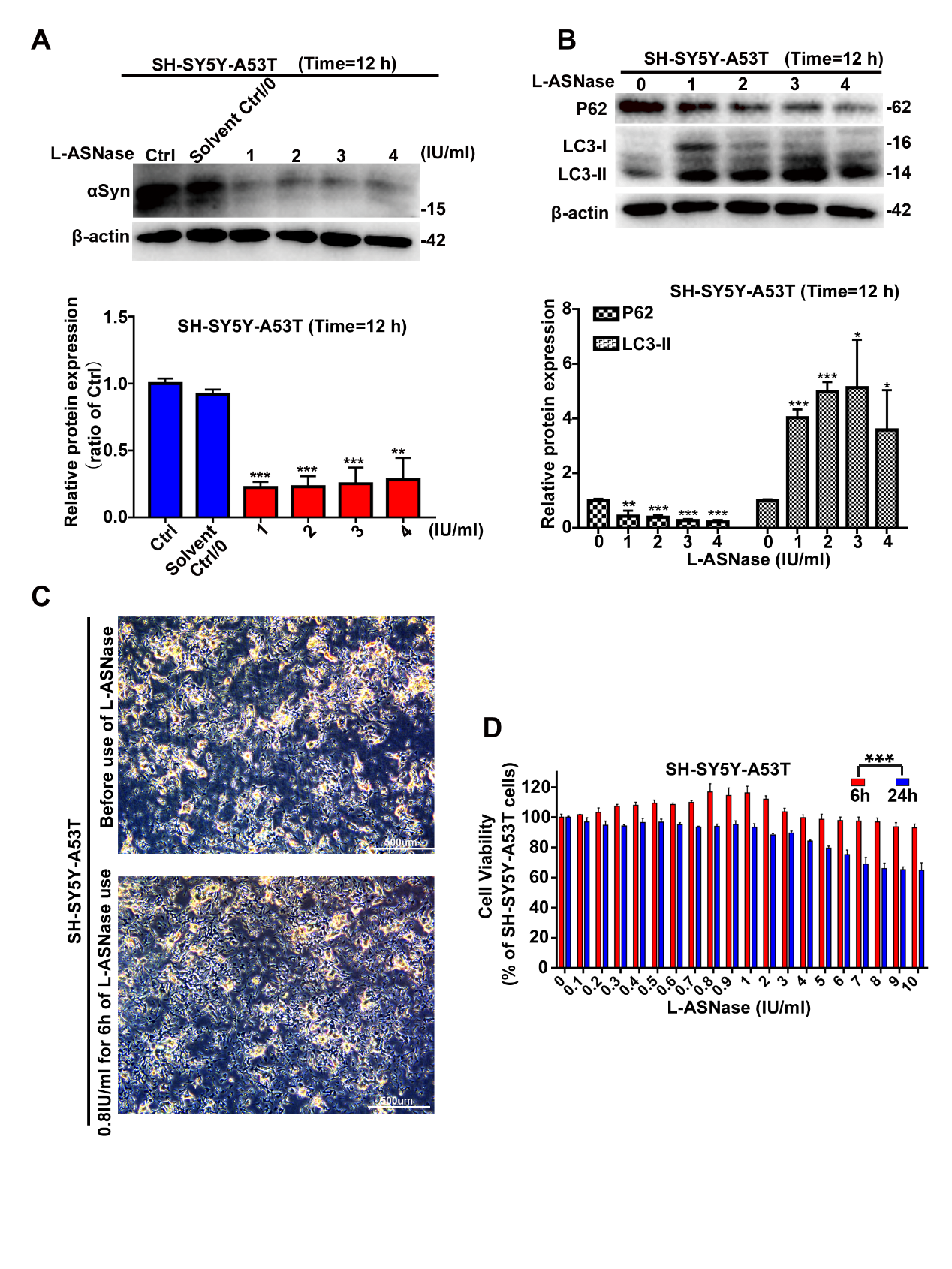
Figure S1**

Figure legend: **(A)** Representative western bolt image indicating the effect of αSyn autophagic degradation ranging from 1 to 4 IU/ ml L-ASNase treated for 12 hours, histogram reflecting the statistics and normalized to control , *** P*<0.01, **** P<*0.001, **(B)** Representative western bolt image showing the increasing expression of LC3-II and P62 induced by 1IU to 4 IU/ ml L-ASNase treated for 12 hours, histogram showing the relative protein expression of P62 and LC3-II in each group vs “SH-SY5Y-A53T cells without L-ASNase treated”, ** P*<0.1,*** P*<0.01, **** P*<0.001. **(C)** Cell morphology of SH-SY5Y-A53T cells under a light microscope (4X) before and after 0.8 IU/ml L-ASNase use. **(D)** The cytotoxicity of L-ASNase of administration concentration points at 0, 0.1, 0.2, 0.3, 0.4, 0.5, 0.6, 0.7, 0.8, 0.9, 1.0, 2.0, 3.0, 4.0, 5.0, 6.0, 7.0, 8.0, 9.0, 10.0 and the administration time points at 6h, 24h were detected, the cytotoxicity of 24 hours treatment was much greater than that of 6 hours treatment, **** P<*0.001. All the above statistics have been repeated three independent experiments, means ± S.D.

**
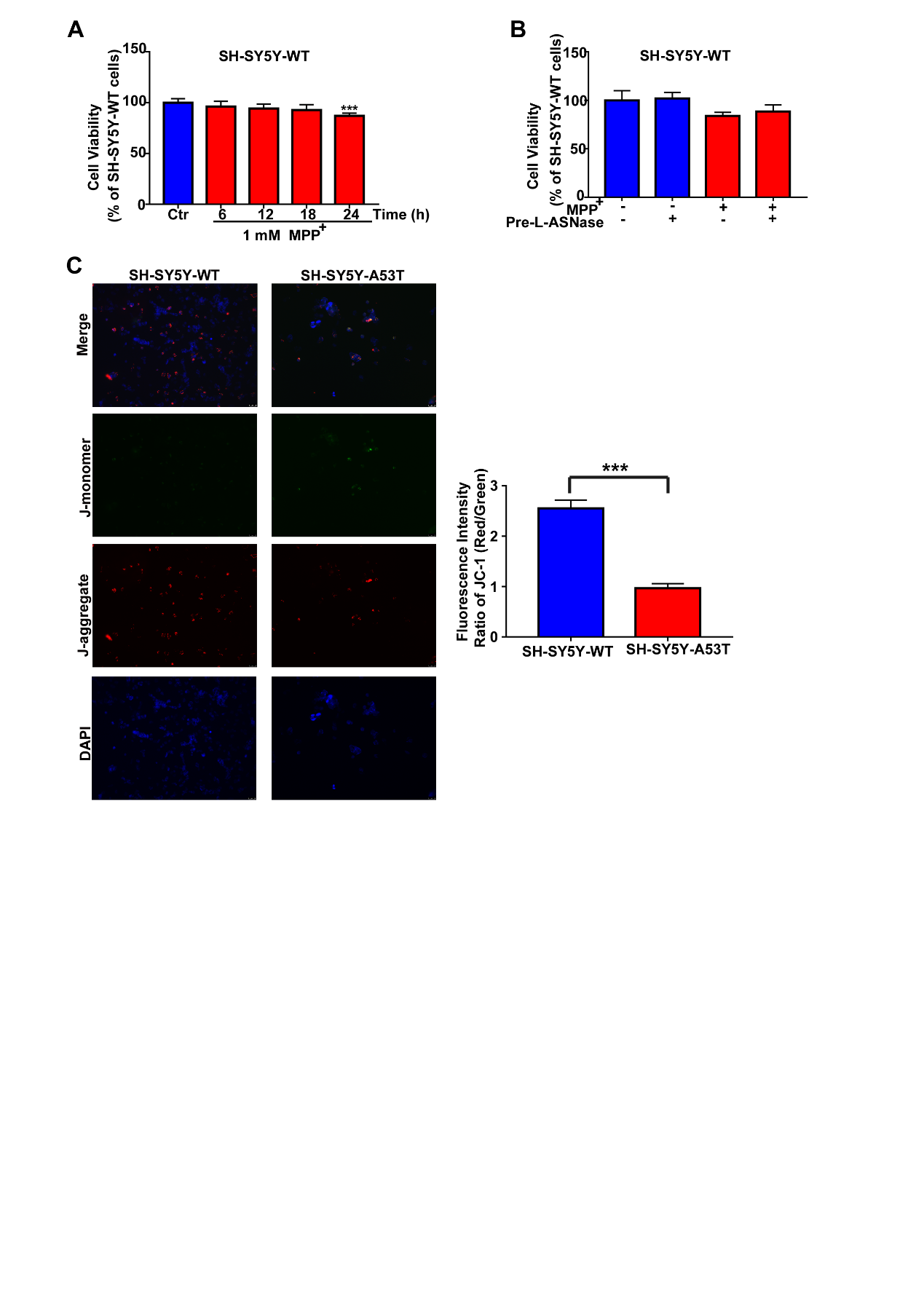
Figure S2**

Figure legend: **(A)** Histogram showing cell viability of wild type (WT) SH-SY5Y cells with 1 mM MPP+ exposed from 0 to 24 h (the time point was set every 6 h) , **** P*<0.001. **(B)** Histogram showing the cell viability of each group, the results of each group were not statistically significant. **(C)** Representative JC-1 staining fluorescence images of SH-SY5Y-A53T and WT SH-SY5Y cells, the histogram showing the statistics between the two groups, **** P*<0.001. All the above data were presented as means ±S.D of three independent experiments.
